# A proposal for uniformity in classification of lymph node stations in esophageal cancer

**DOI:** 10.1093/dote/doab009

**Published:** 2021-03-17

**Authors:** N Schuring, S Matsuda, E R C Hagens, J Sano, S Mayanagi, H Kawakubo, M I van Berge Henegouwen, Y Kitagawa, S S Gisbertz

**Affiliations:** Department of Surgery, Amsterdam UMC, University of Amsterdam, Cancer Center Amsterdam, Amsterdam, The Netherlands; Department of Surgery, Keio University School of Medicine, Tokyo, Japan; Department of Surgery, Amsterdam UMC, University of Amsterdam, Cancer Center Amsterdam, Amsterdam, The Netherlands; Department of Surgery, Keio University School of Medicine, Tokyo, Japan; Department of Surgery, Keio University School of Medicine, Tokyo, Japan; Department of Surgery, Keio University School of Medicine, Tokyo, Japan; Department of Surgery, Amsterdam UMC, University of Amsterdam, Cancer Center Amsterdam, Amsterdam, The Netherlands; Department of Surgery, Keio University School of Medicine, Tokyo, Japan; Department of Surgery, Amsterdam UMC, University of Amsterdam, Cancer Center Amsterdam, Amsterdam, The Netherlands

**Keywords:** Japan Esophageal Society (JES), American Joint Committee on Cancer (AJCC), Union for International Cancer Control (UICC), Esophageal Cancer, Lymph node metastases

## Abstract

The 11^th^ edition of the “Japanese Classification of Esophageal Cancer” by the Japan Esophageal Society (JES) and the 8^th^ edition of the American Joint Committee on Cancer (AJCC)/Union for International Cancer Control (UICC) “Cancer Staging Manual” are two separate classification systems both widely used for the clinical and pathological staging of esophageal cancer. Furthermore, the lymph node stations from these classification systems are combined for research purposes in the multinational TIGER study, which investigates the distribution pattern of lymph node metastases. The existing classification systems greatly differ with regard to number, location and anatomical boundaries of locoregional lymph node stations. The differences in these classifications cause significant heterogeneity in studies on lymph node metastases in esophageal cancer. This makes data interpretation difficult and comparison of studies challenging. In this article, we propose a match for these two commonly used classification systems and additionally for the TIGER study classification, in order to be able to compare results of studies and exchange knowledge and to make steps towards one global uniform classification system for all patients with esophageal cancer.

## Introduction

Lymph node status in esophageal cancer is a significant negative prognostic predictor for overall survival. Lymphadenectomy in esophageal cancer surgery with or without perioperative chemotherapy has been shown to be both of prognostic and therapeutic value, although results following neoadjuvant chemoradiotherapy are conflicting.^1–6^ The possibility of a curative therapeutic strategy depends on the location of lymph node metastases and discussion exists, whether certain lymph node stations are regarded as locoregional or extraregional.^3^^,^^7^^,^^8^ Identifying the distribution pattern of lymph node metastases is important to determine the optimal radiation field if neoadjuvant chemoradiotherapy is applied and to define the optimal extent of lymphadenectomy. Despite the increasing incidence of esophageal cancer, there is no worldwide uniform classification system yet, and no consensus exists on the extent of the radiation field and lymphadenectomy.

The two co-existing systems for classifying lymph node metastases in esophageal cancer are the 11^th^ edition of the “Japanese Classification of Esophageal Cancer” by JES and the 8^th^ edition of the “Cancer Staging Manual” by AJCC/UICC.^7^^,^^8^ These classification systems greatly differ with regard to number, location and anatomical boundaries of locoregional lymph node stations. The differences in these classifications cause significant heterogeneity in studies on lymph node metastases in esophageal cancer, and it makes data interpretation and comparison of these studies challenging, as has been shown in a recent systematic review by Hagens et al.^9^ In this review the distribution pattern of the lymph node metastases in patients with an esophageal adenocarcinoma or squamous cell carcinoma was investigated, and included data of 8952 patients in total from 14 different studies. No formal meta-analysis could be performed since there was great variation in how lymph node stations were defined.^9^ In addition to scientific difficulties, differences in classifying lymph node metastases do not contribute to uniform treatment protocols.

The TIGER study [NCT 03222895] investigates the distribution of lymph node metastasis in patients with esophageal cancer.^10^ In this study, renown esophageal cancer centers from all over the world are collaborating and for study purposes the two existing classification systems were combined.

In this article, we propose a match for the two commonly used classification systems and additionally for the TIGER study classification. This proposal may contribute to the exchange of knowledge, comparison of study results and in making steps towards one global uniform classification system for all patients with esophageal cancer.

### The esophageal lymphatic system

The esophagus is a muscular tube, connecting the hypopharynx with the stomach, thereby crossing three different anatomical compartments in the human body: the neck, the mediastinum and the upper abdomen. Its anatomical position ensures close contact with surrounding structures and organs, such as the diaphragm, the pericardium, the aorta, the trachea, the vertebrae and the pleurae.^7^^,^^8^ When describing the location of a primary tumor the esophagus is divided into a cervical (upper), thoracic (upper, middle or lower) and abdominal (abdominal, esophagogastric junction) part. In all three compartments, the esophagus has vascular and lymphatic connections. The lymphatic system surrounding the esophagus is complexly organized and contributes to the multidirectional spread of metastatic cells in lymph nodes in all three compartments, as has been shown in a prospective study on sentinel lymph nodes in early esophageal squamous cell carcinoma.^11^ In this study, radio-guided detection was used to identify sentinel lymph nodes and the results showed that successfully identified sentinel lymph nodes were widely distributed from cervical to abdominal areas with an average of 4.7 sentinel lymph nodes.^11^ Similar results were found in a Western pilot-study in patients with an early distal esophageal adenocarcinoma.^12^ In this study, a median of 5 (IQR: 3–10) sentinel lymph nodes were identified by endoscopic gamma probe, at 3 locations (median; IQR:2–5) ranging from high right paratracheal to the celiac trunc.^12^

The esophageal wall consists of different layers: on the inside the mucosa, then the submucosa, the muscularis propria and the adventitia. The lymphatic drainage is mainly located in the submucosa, but lymphatic channels also have minor branches in the lamina propria of the mucosa, which can cause lymphatic metastases at an early stage of esophageal cancer.^3^ Another observed phenomenon is the occurrence of skip metastases; metastasis in distant lymph nodes without positive lymph nodes in the direct surrounding of the primary tumor. Skip metastases can occur as a consequence of the presence of lymphatic branches in the submucosa and even in the lamina propria, consisting of a dense network, with lymph flow both intramurally and longitudinally and directed both cranially and caudally. This may cause lymph node metastases in unpredictable locations distributed in caudal, cranial or in lateral direction.^7–9^

### A short history on classification systems of esophageal cancer

#### Japanese Classification of Esophageal Cancer

**Fig. 1:**
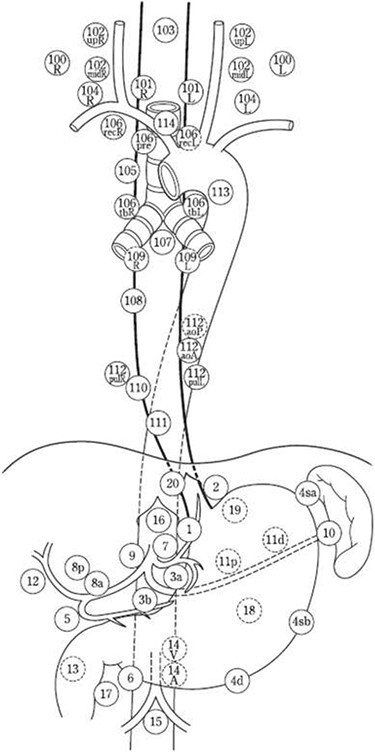
JES classification (11th edition) station numbers of regional lymph nodes *[This figure is re-used. The original source:* Japanese Classification of Esophageal Cancer, 11th Edition by the Japan Esophageal Society. (*CC BY 4.0)]^L1^*.

The first edition *“The Guidelines for the Clinical and Pathologic Studies on Carcinoma of the Esophagus”* was originally published in 1969 by the Japanese Society for Esophageal Diseases (JSED). In 2003, the name of the society was changed into the Japanese Esophageal society (JES). Since then the JES has published successive editions of “the Japanese Classification of Esophageal Cancer” with revisions and modifications for changes in diagnostics and treatment over time. The latest, 11^th^ edition, was published in October 2015. This classification system is very detailed, both in the abdomen (adapted from the Japanese gastric cancer classification), mediastinum and neck. The system, numbers lymph node stations from 100–104 (neck), 105–114 (mediastinum) and from 1–20 (abdomen) and also contains lymph node grouping (N1 - N4 lymph node groups) according to inter alia the primary tumor location (cervical, upper thoracic, middle thoracic, lower thoracic and abdominal; *figure 1*). Recommendations are provided which lymph node groups to resect per primary tumor location by the JES guidelines.^13^ With every successive edition the JES revised the N-grouping by collecting registry data from most of the esophageal cancer centers in Japan, considering the rate of lymph node metastases and prognosis of each lymph node station with regard to primary tumor location. An efficacy index is calculated, wherein resection of a lymph node station with a high index is more effective for prognosis than resection of a lymph node station with a low index.^14^ N1 lymph nodes have the highest efficacy for prognosis when resected and N4 lymph nodes the lowest.^14^

#### AJCC Cancer Staging Manual

**Fig. 2:**
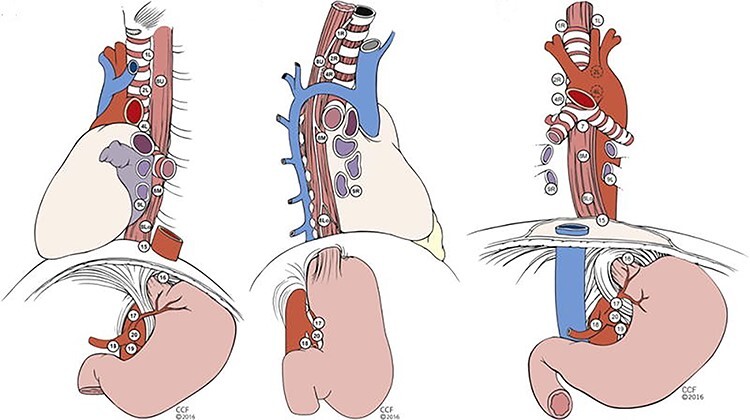
AJCC classification (8th edition) Regional lymph node maps for esophageal cancer [Re-used from “*Cancer of the Esophagus and Esophagogastric Junction: An eighth edition staging Primer by Thomas W. Rice et al. published October 31, 2016* with permission from Elsevier. License number 4891390623081].

The TNM staging system in the “Cancer Staging Manual” was developed by the AJCC and the UICC. This is a classification system for cancer based on three main factors: tumor invasion depth (T), lymph node involvement (N) and distant metastases (M). The first classification for esophagus and esophagogastric junction cancer staging by the AJCC was published in the first edition of the staging manual in 1977. The first cancer staging manual based on TNM cancer staging was published in 1968 by the UICC. In 1988, the UICC and the AJCC esophageal cancer staging guidelines were unified. The latest, 8^th^ edition is effective since the beginning of 2017. In this classification system, the lymph node stations in the thorax and abdomen are numbered from 1–20. Except for the lower cervical paratracheal lymph nodes, the cervical lymph nodes are not represented in this classification, and reference is made to the head and neck chapter, with level VI and VII from this classification regarded as locoregional disease.^8^ N-status is calculated by the number of positive lymph nodes and is subdivided into N0 to N3 (*figure 2*).^3^^,^^8^

#### TIGER lymph node classification system

**Fig. 3:**
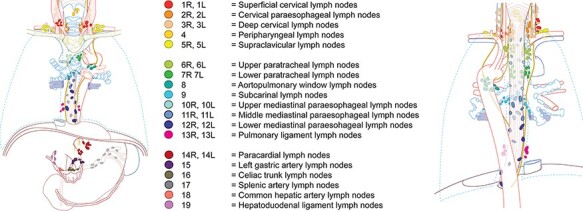
TIGER classification (1^st^ edition) station numbers and naming of regional lymph nodes [This figure is re-used. The original source: Distribution of lymph node metastases in esophageal carcinoma [TIGER study]: study protocol of a multinational observational study by Hagens et al. Published in BMC cancer 2019. (CC0 1.0)]^L2^ LINKS. L1. https://creativecommons.org/licenses/by/4.0/legalcode L2. https://creativecommons.org/publicdomain/zero/1.0/legalcode.

The first edition of the TIGER classification for lymph node stations in esophageal cancer was designed in 2016 and published in 2019 in the context of the TIGER study.^10^ This is an international observational cohort study investigating the distribution of lymph node metastases in esophageal cancer with surgeons and pathologists from over 50 renowned esophageal cancer centers from all over the world collaborating. In this classification the AJCC 8^th^ edition and the JES 11^th^ edition are combined for research purposes and lymph node stations are numbered 1–5 (neck), 6–13 (mediastinum) and 14–19 (abdomen) (*figure 3*  *and table S1*).^10^ After obtaining and processing the data of the TIGER study, the study results may contribute to the global use of one classification system for all esophageal cancer patients. In addition, with the study results an Efficacy Index of each lymph node station can be calculated and regional and non-regional lymph nodes can be defined. This may offer support for national and international guidelines on extent of lymphadenectomy and on which lymph node stations should be resected with regard to different tumor characteristics.

### Differences between the existing classification systems

The JES and the AJCC classification both consist of three main categories; T, N and M with the largest difference in the N-category.^7^^,^^8^ Although the JES strived to more uniformity with the AJCC classification in the latest edition, this was, due to insurmountable differences, not possible for the classification of lymph node stations. The main differences between the systems in lymph node stations are not only the given numbers and names, but especially the anatomical boundaries of the lymph node stations. The largest differences are observed in the neck and upper mediastinum. In the upper mediastinum for example, the JES follows the course of the recurrent laryngeal nerves (station 106recL/R) and the AJCC divides these in upper and lower paratracheal lymph node stations (station 2 L/R and 4 L/R) (*figure 1*  *and*  *2*). In the neck, the AJCC consists of 7 levels and the JES of 5 main groups subdivided in 12 subgroups with different anatomical boundaries for groups/levels of the two classifications. In addition, in the AJCC classification the N-category is defined by the number and not location of metastatic regional lymph nodes (station 1–20). Cervical lymph nodes, except for station 1 and level VI and VII (Head and Neck AJCC cancer staging) are not considered as locoregional lymph node metastases but as extraregional lymph node metastases and thus as M-disease, independent of the location or the histology of the primary tumor. In the JES system, regional lymph nodes (100–104 neck, 105–114 mediastinum, 1–20 abdomen) are grouped into N1–N4 lymph node groups in five different patterns according to the primary tumor location (cervical, upper thoracic, middle thoracic, lower thoracic and abdominal). Whether lymph node metastases are considered as locoregional (and should be resected as part of standard lymphadenectomy) depends on primary tumor location, and was established by the incidence rate of lymph node metastases and prognosis for each lymph node station with regard to location of the primary tumor. Not only do the two classification systems differ in anatomical boundaries of lymph node stations, they also differ in what are regarded as loco-regional and extra-regional lymph node stations. As described, in the JES, this depends on primary tumor location, in the AJCC, this distinction is not made, although this information may have major prognostic impact. This is also one of the main research questions of the TIGER study.

There are several explanations for the differences between these two systems. In Asia, the majority of the esophageal cancer patients is diagnosed with a squamous cell carcinoma, while in Europe and North America an adenocarcinoma is the predominant tumor type.^15^^,^^16^ The primary tumor location of squamous cell cancer in the esophagus varies from cervical to abdominal, while the adenocarcinoma is located in the distal esophagus or at the gastro-esophageal junction. Cervical or upper mediastinal lymph node metastases are therefore less frequently seen, and often only resected by Western surgeons on indication, while a 3-field lymphadenectomy is standard practice in the East.^17^^,^^18^ In addition, the AJCC esophageal cancer staging was adapted from the lung cancer AJCC staging, and combined with part of the and head and neck cancer AJCC staging, because studies on distribution of lymph node metastases in esophageal cancer are scarce. The evidence for the AJCC 8^th^ edition came from the WEC database, in which the location of lymph node metastases was not considered, only the number of positive lymph nodes.^8^ The JES abdominal lymph node stations are adopted from the Japanese Gastric Cancer staging, however, the mediastinal and cervical part is specifically designed for esophageal cancer and based on large studies of location of lymph node metastases from Japan.^7^

### A common language

One uniform classification system will be beneficial for studying the behavior of esophageal cancer and specifically the incidence and location of lymph node metastases. The differences between the two official classification systems have been addressed before.^19^ In this study, investigating the efficacy of lymph node dissection by area, the comparison of the two classification systems was not performed for all lymph node stations. Another attempt was made with the TIGER study classification, which combined the JES and the AJCC classification. This classification is an endeavor to reach global consensus on the use of one classification system for all patients with esophageal cancer. However, this classification also does not solve all problems. Previous studies on lymph node metastases in esophageal cancer have been using either the JES or the AJCC classification, or some other anatomical grouping, and consequently, cannot be directly compared or calculated with. Therefore, we propose a match for these two commonly used classification systems and additionally for the TIGER study classification as shown in table 1.

**Table 1:** Proposed match of the AJCC, JES and TIGER classification system for lymph node stations in esophageal cancer (continues on next page)

JES (11^th^ edition)		AJCC (8^th^ edition)		TIGER (1^st^ edition)	
Number	Name	Number	Name	Number	Name
***Cervical Lymph node stations***					
		IA^*^	Submental lymph nodes		
		VII^*^	Superior mediastinal lymph nodes		
100 spf	Superficial cervical lymph nodes			1	Superficial cervical lymph nodes
100 sm	Submandibular lymph nodes	IB^*^	Submandibular lymph nodes	1	Superficial cervical lymph nodes
100 tr	Cervical pretracheal lymph nodes	VI^*^	Anterior compartment lymph nodes	1	Superficial cervical lymph nodes
100 ac	Accessory nerve lymph nodes	VA & VB^*^	Posterior triangle lymph nodes	1	Superficial cervical lymph nodes
101	Cervical paraesophageal lymph nodes	1 R/L (IV^*^)	Lower cervical paratracheal lymph nodes	2	Cervical paraesophageal lymph nodes
102 up	Upper deep cervical lymph nodes	IIB^*^	Upper jugular lymph nodes	3	Deep cervical lymph nodes
102 mid	Middle deep cervical lymph nodes	III^*^	Middle jugular lymph nodes	3	Deep cervical lymph nodes
103	Peripharyngeal lymph nodes	IIA & III^*^	Upper jugular & Middle jugular lymph nodes	4	Peripharyngeal lymph nodes
104	Supraclavicular lymph nodes	IV & VB^*^	Lower jugular & posterior triangle lymph nodes	5	Supraclavicular lymph nodes
***Thoracic Lymph Node stations***					
105	Upper thoracic paraesophageal lymph nodes	8up	Upper thoracic paraesophageal lymph nodes	10	Upper mediastinal paraesophageal lymph nodes
106 recL	Left recurrent nerve lymph nodes	2 L	Left upper paratracheal lymph nodes	6 L	Left upper paratracheal lymph nodes
106 recR	Right recurrent nerve lymph nodes	2 R	Right upper paratracheal lymph nodes	6 R	Right upper paratracheal lymph nodes
106 pre	Pretracheal lymph nodes	4 R	Right lower paratracheal lymph nodes	7 R	Right lower paratracheal lymph nodes
106 tbL	Tracheobronchial lymph nodes	4 L	Left lower paratracheal lymph nodes	7 L	Left lower paratracheal lymph nodes
106 tbR	Right tracheobronchial lymph nodes	4 R	Right lower paratracheal lymph nodes	7 R	Right lower paratracheal lymph nodes
107	Subcarinal lymph nodes	7	Subcarinal lymph nodes	9	Subcarinal lymph nodes
108	Middle thoracic paraesophageal lymph nodes	8 m	Middle thoracic paraesophageal lymph nodes	11	Middle mediastinal paraesophageal lymph nodes
109	Main bronchus lymph nodes	10*®*	Tracheobronchial lymph nodes	9	Subcarinal lymph nodes
110	Lower thoracic paraesophageal lymph nodes	8lo	Lower thoracic paraesophageal lymph nodes	12	Lower mediastinal paraesophageal lymph nodes
111	Supradiaphragmatic lymph nodes	15	Diaphragmatic lymph nodes	12	Lower mediastinal paraesophageal lymph nodes
112 ao(A/P)	thoracic paraaortic lymph nodes (Anterior/Posterior)	8 m & 8lo	Middle thoracic paraesophageal lymph nodes & Lower thoracic paraesophageal lymph nodes	11 & 12	Middle mediastinal paraesophageal lymph nodes /Lower mediastinal paraesophageal lymph nodes
112 pul	Pulmonary ligament lymph nodes	9 R/L	Pulmonary ligament lymph nodes	13 R/L	Pulmonary ligament lymph nodes (L/R)
113	Ligamentum arteriosum lymph nodes (Botallo lymph nodes)	5^#^	Aortopulmonary lymph nodes	8	Aortopulmonary window lymph nodes
114	Anterior mediastinal lymph nodes				
***Abdominal Lymph node stations***					
1	Right paracardial lymph nodes	16	Paracardial lymph nodes	14 R	Right paracardia lymph nodes
2	Left paracardial lymph nodes	16	Paracardial lymph nodes	14 L	Left paracardia lymph nodes
3 a	Lesser curvature lymph nodes	17	Left gastric lymph nodes	15	Left gastric lymph nodes
3 b	Lesser curvature lymph nodes				
4 sa	Lymph nodes along the short gastric vessels				
4 sb	Lymph nodes along the left gastroepiploic artery				
4 d	Lymph nodes along the right gastroepiploic artery				
5	Suprapyloric lymph nodes				
6	Infrapyloric lymph nodes				
7	Lymph nodes along the left gastric artery	17	Left gastric lymph nodes	15	Left gastric lymph nodes
8 a	Lymph nodes along the common hepatic artery (anterosuperior group)	18	Common hepatic lymph nodes	18	Common hepatic artery lymph nodes
8 p	Lymph nodes along the common hepatic artery (posterior group)	18	Common hepatic lymph nodes	18	Common hepatic artery lymph nodes
9	Lymph nodes along the celiac artery	20	Celiac lymph nodes	16	Celiac trunk lymph nodes
10	Lymph nodes at the splenic hilum			17	Splenic artery lymph nodes
11 p	Lymph nodes along the proximal splenic artery	19	Splenic lymph nodes	17	Splenic artery lymph nodes
11 d	Lymph nodes along the distal splenic artery	19	Splenic lymph nodes	17	Splenic artery lymph nodes
12	Lymph nodes in the hepatoduodenal ligament			19	Hepatoduodenal ligament lymph nodes
13	Lymph nodes on the posterior surface of the pancreatic head				
14 A	Lymph nodes along the superior mesenteric artery				
14 V	Lymph nodes along the superior mesenteric vein				
15	Lymph nodes along the middle colic artery				
16 a1	Lymph nodes in the aortic hiatus				
16 a2	Lymph nodes around the abdominal aorta				
16 b1	Lymph nodes around the abdominal aorta				
16 b2	Lymph nodes around the abdominal aorta				
17	Lymph nodes on the anterior surface of the pancreatic head				
18	Lymph nodes along the inferior margin of the pancreas				
19	Infradiaphragmatic lymph nodes	16	Paracardial lymph nodes	14 R/L	Paracardia lymph nodes
20	Lymph nodes in the esophageal hiatus of the diaphragm	16	Paracardial lymph nodes	14 R/L	Paracardia lymph nodes

*® AJCC Lung cancer staging (8^th^ edition)*

* AJCC Head and neck cancer staging (8^th^ edition)

^#^ *AJCC Cancer staging manual: Esophagus and esophagogastric junction (7^th^ edition)*

### Discussion and future perspectives

This article describes the history of and the heterogeneity in the two most commonly used classification systems for lymph node staging in esophageal cancer. Addressing these differences resulted in a proposed match for those classifications and additionally for the TIGER classification. This proposal may contribute to the development and implementation of one worldwide uniform classification system for all esophageal cancer patients.

Some studies, mostly from the East, have investigated the distribution of lymph node metastases in esophageal cancer. Consequently, most of these studies are on squamous cell carcinoma, and adenocarcinoma, which is more frequently diagnosed in the West, is less well studied.^9^ In addition, lymphadenectomy in the East is usually more extended compared to the West, especially in the neck and upper mediastinum, even though it has been shown that lymphadenectomy in esophageal cancer surgery has both prognostic and therapeutic value.^1–6^ This is especially true for patients following primary surgery or perioperative chemotherapy.^5^ Results following neoadjuvant chemoradiotherapy remain conflicting.^2^^,^^4^

A recent prospective nationwide study from Japan, investigated the distribution of lymph node metastases in gastro-esophageal junction cancer.^20^ Even though this is a study from the East, most of the included patients were diagnosed with an adenocarcinoma. The study results show, that in the case of a gastro-esophageal junction adenocarcinoma with >3 cm esophageal involvement or a squamous cell carcinoma, the lymph node metastases rate of at least 1 upper mediastinal lymph node station was 6.1%, and of the middle mediastinal lymph node stations 7.1%.^20^ The authors published a flow chart for surgical approach in relation to esophageal involvement of the gastro-esophageal junction tumor, and propose a right transthoracic esophagectomy if the gastro-esophageal junction tumor invades the esophagus >4 cm. The TIGER study investigates the distribution of lymph node metastases in esophageal cancer in many different tumor locations, in different histology types and invasion depth and in patients who did or did not receive (neo)adjuvant therapy. In addition, the number of resected lymph nodes, lymph node metastases and the efficacy index will be recorded and calculated. Furthermore, this study will investigate the occurrence of lymph node metastases in relation to the radiation field if radiotherapy is applied and the location and patterns of any recurrent disease.^10^ Ultimately, the data of the TIGER study will not only contribute to our knowledge of this unpredictable disseminating disease but may also offer support to which lymph node stations should be regarded as regional or non-regional depending on tumor specific characteristics such as tumor location, histology, invasion depth and tumor length and type of neoadjuvant therapy. In addition, the Efficacy Index of each lymph node station can be calculated, which may contribute to guidelines on extent of lymphadenectomy and on which lymph nodes should be resected with regard to specific tumor characteristics.

A few limitations of this proposal have to be addressed. The proposed match is made by a surgical collaboration of a university hospital from the East and one from the West. This match has not yet been consented by other experts, not yet been studied and not yet been validated. In addition, since not all lymph node stations can be exactly matched because of slightly differing anatomical boundaries, this proposed match could lead to some simplification. As a consequence, after matching, the prognostic value of lymph node metastases in a specific station in one classification, may encompass a larger or slightly different anatomical area in the other classification.

To gain worldwide consensus on classifying lymph node metastases and lymphadenectomy, more research is needed. Results from the before mentioned TIGER study will not only provide more evidence for such a uniform classification, but this worldwide collaboration between East and West could also form the foundation to reach global consensus and may contribute to the use of one classification system. When these data are validated, new staging and treatment guidelines can be implemented, including a recommended extend of the radiation field and lymphadenectomy based on histology, T-stage, affected lymph node stations and primary tumor location. Additionally, this approach will facilitate future research and, eventually, patients with esophageal cancer will benefit from this.

In conclusion, at this moment there is no uniform classification system for lymph node metastases in esophageal carcinoma and therefore there is no consensus on lymphadenectomy in patients with esophageal cancer. This article proposes a match for the two established classifications for lymph node stations in esophageal cancer; this will contribute to uniformity and better comparison of studies on patients with esophageal cancer. The data of the TIGER study may contribute to global consensus for one classification system for all patients with esophageal cancer.

## Disclosure

MIvBH reports grants from Olympus and Stryker; personal fees from Johnson and Johnson, Medtronic, Mylan and Alesi Surgical. All fees paid to institution outside the submitted work.

YK reports grants from Taiho Pharmaceutical, Chugai Pharmaceutical, Yakult Honsha, Daiichi Sankyo, Merck Serono, Asahi Kasei, EA Pharma, Otsuka Pharmaceutical, Takeda Pharmaceutical, Otsuka Pharmaceutical Factory, Shionogi, Kaken Pharmaceutical, Kowa Pharmaceutical, Astellas Pharma, Medicon, Dainippon Sumitomo Pharma, Taisho Toyama Pharmaceutical, Kyouwa Hakkou Kirin, Pfizer Japan, Ono Pharmaceutical, Nihon Pharmaceutical, Japan Blood Products Organization Medtronic Japan, Sanofi K.K., Eisai, Tsumura & Co., Ltd., KCI Licensing, Abbott Japan, FUJIFILM, and Toyama Chemical. Moreover, he received lecture fees from Asahi Kasei KASEI, Taiho pharmaceutical, Chugai Pharmaceutical, EA Pharma, Yakult Honsha, Otsuka Pharmaceutical, Otsuka Pharmaceutical Factory, Shionogi & Co, Astellas Pharma, Dainippon sumitomo pharma, Taisho Toyama Pharmaceutical, Ono Pharmaceutical, Nihon Pharmaceutical, Sanofi K.K., Eisai, Kaken Pharmaceutical. All fees paid to institution outside the submitted work.

## Supplementary Material

Supplementary_file_1_Disclosure_doab009

S1_table_Final_doab009

## References

[1] Miyata H, Sugimura K, Yamasaki M et al. Clinical impact of the location of lymph node metastases after neoadjuvant chemotherapy for middle and lower thoracic esophageal cancer. Ann Surg Oncol 2019 (Jan); 26(1): 200–8. 30374924 10.1245/s10434-018-6946-z

[2] Visser E, Markar S R, Ruurda J P, Hanna G B, van Hillegersberg R. Prognostic value of lymph node yield on overall survival in esophageal cancer patients: a systematic review and meta-analysis. Ann Surg 2019 (Feb); 269(2): 261–8. 29794846 10.1097/SLA.0000000000002824

[3] Rice T W, Ishwaran H, Ferguson M K, Blackstone E H, Goldstraw P. Cancer of the esophagus and esophagogastric junction: an eight edition staging primer. J Thorac Oncol 2017 (Jan); 12(1): 36–42. 27810391 10.1016/j.jtho.2016.10.016 PMC5591443

[4] Oppedijk V, van der Gaast A, van Lanschot J J B et al. Patterns of recurrence after surgery alone versus preoperative chemoradiotherapy and surgery in the CROSS trials. J Clin Oncol 2014 (Feb); 32: 385–91. 24419108 10.1200/JCO.2013.51.2186

[5] Markar S R, Noordman B J, Mackenzie H et al. Multimodality treatment for esophageal adenocarcinoma: multi-center propensity-score matched study. Ann Oncol 2017 (Mar 1); 28(3): 519–27. 28039180 10.1093/annonc/mdw560 PMC5391716

[6] Talsma K A, Shapiro J, Looman C W N, van Hagen P et al. Lymph node retrieval during esophagectomy with and without neoadjuvant chemoradiotherapy: prognostic and therapeutic impact on survival. Ann Surg 2014 (Nov); 260(5): 786–92. 25379850 10.1097/SLA.0000000000000965

[7] Japan Esophageal Society . Japanese classification of Esophageal Cancer, 11th edition: part I and part II and III. Esophagus 2017; 14: 1–36 37–65. 28111535 10.1007/s10388-016-0551-7 PMC5222932

[8] Amin M B, Gress D M, Meyer Vega L R et al. AJCC Cancer Staging Manual, Eight Edition. New York: Springer 2017.

[9] Hagens E R C, van Berge Henegouwen M I, Gisbertz S S. Distribution of lymph node metastases in esophageal carcinoma patients undergoing upfront surgery: a systematic review. Cancer (basel) 2020 (Jun 16); 12(6): LE1592. 10.3390/cancers12061592 PMC7352338 32560226

[10] Hagens E R C, van Berge Henegouwen M I, van Sandick J W et al. Distribution of lymph node metastases in Esophageal carcinoma [TIGER study]: study protocol of a multinational observational study. BMC Cancer 2019 (Jul); 19(1): 662. 31272485 10.1186/s12885-019-5761-7 PMC6610993

[11] Takuechi H, Fujii H, Ando N et al. Ann Surg 2009 (May); 249(5): 757–63. 19387329 10.1097/SLA.0b013e3181a38e89

[12] Kunzli H T, van Berge Henegouwen M I, Gisbertz S S et al. Pilot-study on the feasibility of sentinel node navigation surgery in combination with thoracolaparoscopic lymphadenectomy without esophagectomy in early esophageal adenocarcinoma patients. Diseases of the Esophagus 2017 (Nov); 30(11): 1–8. 10.1093/dote/dox097 28881907

[13] Kitagawa Y, Uno T, Oyama T et al. Esophageal cancer practice guidelines 2017 edited by the Japan esophageal society: part 2. Esophagus 2019; 16(1): 24–43. 10.1007/s10388-018-0641-9 PMC6510883 30171413

[14] Udagawa H, Ueno M, Shinohara H et al. The Importance of Grouping of Lymph Node Stations and Rationale of Three-Field Lymphoadenectomy. J Surg Oncol 2012;106(6):742–7. 10.1002/jso.23122 22504922

[15] Malhotra G K, Yanala U, Ravipati A, Follet M, Vijayakumar M, Are C. Global trends in esophageal cancer. J Surg Oncol United States 2017 (Apr); 115: 564–79. 10.1002/jso.24592 28320055

[16] Pennathur A, Gibson M K, Jobe B A, Luketich J D. Oesophageal carcinoma. Lancet Elsevier Ltd 2013; 381: 400–12. 10.1016/S0140-6736(12)60643-6 23374478

[17] Akutsu Y, Matsubara H. Lymph node dissection for esophageal cancer. Gen Thorac Cardiovasc Surg 2013; 61(7): 397–401. 23529259 10.1007/s11748-013-0237-1

[18] Matsuda S, Takuechi H, Kawakubo H, Kitagawa Y J. Three-field lymph node dissection in esophageal cancer surgery. Thorac Dis 2017 (Jul); 9(Suppl 8): S731–40. 10.21037/jtd.2017.03.171 PMC5538994 28815069

[19] Tachimori Y, Ozawa S, Numasaki H et al. Efficacy of lymph node dissection by node zones according to tumor location for esophageal squamous cell carcinoma. Esophagus 2016; 13: 1–7. 26752982 10.1007/s10388-015-0515-3 PMC4698372

[20] Kurokowa Y, Takeuchi H, Doki Y et al. Mapping of lymph node metastasis from esophagogastric junction tumors: a prospective nationwide multicenter study. Ann Sur 2019; Volume publish ahead of print. 10.1097/SLA.0000000000003499 31404008

