# Supplementary material for: A proposal for uniformity in classification of lymph node stations in esophageal cancer
Source: Dis Esophagus. 2021 Mar 17;34(10):doab009. doi: 10.1093/dote/doab009 (PMC8503476; doi:10.1093/dote/doab009)
Supplement: Supplementary_file_1_Disclosure_doab009 [file supplementary_file_1_disclosure_doab009.docx]

**Supplementary file 1.**

**Disclosure**

MIvBH reports grants from Olympus and Stryker; personal fees from Johnson and Johnson, Medtronic, Mylan and Alesi Surgical. All fees paid to institution outside the submitted work.

YK reports grants from Taiho Pharmaceutical, Chugai Pharmaceutical, Yakult Honsha, Daiichi Sankyo, Merck Serono, Asahi Kasei, EA Pharma, Otsuka Pharmaceutical, Takeda Pharmaceutical, Otsuka Pharmaceutical Factory, Shionogi, Kaken Pharmaceutical, Kowa Pharmaceutical, Astellas Pharma, Medicon, Dainippon Sumitomo Pharma, Taisho Toyama Pharmaceutical, Kyouwa Hakkou Kirin, Pfizer Japan, Ono Pharmaceutical, Nihon Pharmaceutical, Japan Blood Products Organization Medtronic Japan, Sanofi K.K., Eisai, Tsumura & Co., Ltd., KCI Licensing, Abbott Japan, FUJIFILM, and Toyama Chemical. Moreover, he received lecture fees from Asahi Kasei KASEI, Taiho pharmaceutical, Chugai Pharmaceutical, EA Pharma, Yakult Honsha, Otsuka Pharmaceutical, Otsuka Pharmaceutical Factory, Shionogi & Co, Astellas Pharma, Dainippon sumitomo pharma, Taisho Toyama Pharmaceutical, Ono Pharmaceutical, Nihon Pharmaceutical, Sanofi K.K., Eisai, Kaken Pharmaceutical. All fees paid to institution outside the submitted work.

The remaining authors declare no conflicts of interest.
