# Supplementary material for: A proposal for uniformity in classification of lymph node stations in esophageal cancer
Source: Dis Esophagus. 2021 Mar 17;34(10):doab009. doi: 10.1093/dote/doab009 (PMC8503476; doi:10.1093/dote/doab009)
Supplement: S1_table_Final_doab009 [file s1_table_final_doab009.pdf]

S1 table. *TIGER classification (1st edition) numbers, names and anatomical boundaries. (continues on next page)*

TIGER (1st edition)	
**Number and Name**	**Borders lymph node station**
**Cervical & supraclavicular lymph nodes**	
**1(L/R)** Superficial cervical lymph nodes	• Lymph nodes located along the external jugular veins and anterior jugular veins beneath the superficial cervical fascia.<br>• Lymph nodes located around the submandibular glands and parotid glands, and anterior to the mylohyoid muscle.<br>• Lymph nodes located in the pretracheal fatty tissue, extending from the hyoid bone superiorly, to the left brachiocephalic vein inferiorly, including the prethyroidal lymph nodes and the prelaryngeal lymph nodes.<br>• Lymph nodes located along the accessory nerve(s), and anterior to the trapezius muscle.
**2(L/R)** Cervical paraesophageal lymph nodes	• Lymph nodes located around the cervical esophagus, including lymph nodes located along the recurrent laryngeal nerve and the cervical paratracheal lymph nodes. The lateral boundary is the medial border of the carotid sheath.
**3(L/R)** Deep cervical lymph nodes	• Lymph nodes located around the internal jugular vein and the common carotid artery.<br>• Lymph nodes located from the caudal border of the digastric muscle superior to the carotid artery bifurcation.<br>• Lymph nodes located from the carotid artery bifurcation superiorly to the lower border of the cricoid cartilage inferiorly.
**4** Peripharyngeal lymph nodes	• Lymph nodes located medial to the carotid sheath, extending from the caudal border of the digastric muscle superiorly to the lower border of the cricoid cartilage inferiorly. Postpharyngeal and parapharyngeal lymph nodes are included.
**5(L/R)** Supraclavicular lymph nodes	• Lymph nodes located in the supraclavicular fossa, extending from the lower border of the cricoid cartilage superiorly, to the clavicle inferiorly, including the lower internal deep cervical lymph nodes. The medial boundary is the medial border of the carotid sheath.
**Upper thoracic lymph nodes**	
**6L** Left upper paratracheal lymph nodes	• Lymph nodes located around the upper thoracic esophagus. Lymph nodes located along the anterior and lateral wall of the thoracic trachea until the upper margin of the aortic arch. Lymph nodes located along the left recurrent laryngeal nerve in the mediastinum. The superior boundary is drawn from the cephalic border of the left subclavian artery to the suprasternal notch.
**6R** Right upper paratracheal lymph nodes	• Lymph nodes located around the upper thoracic esophagus posterior to the right vagal nerve.<br>• Lymph nodes located along the anterior and lateral wall of the thoracic trachea until the level of the right vagal nerve.<br>• Lymph nodes located along the right recurrent laryngeal nerve in the mediastinum. The superior boundary is drawn from the cephalic border of the right subclavian artery to the suprasternal notch.
**7L** Left lower paratracheal lymph nodes	• Lymph nodes located in the tracheobronchial angle and located along the anterior and lateral wall of the thoracic trachea. Lymph nodes located along the azygos vein arch and the right bronchial artery are included. Lymph nodes along the proximal part of the left recurrent laryngeal nerve along the aortic arch are also included. The superior boundary is the inferior wall of the aortic arch, and the lymph nodes are located in the area surrounded by the medial wall of the aortic arch.
**7R** Right lower paratracheal lymph nodes	• Lymph nodes located in the tracheobronchial angle and located along the anterior and lateral wall of the thoracic trachea. The superior boundary is the vagal nerve, the ventral boundary the superior caval vein.
**8** Aortopulmonary window lymph nodes	• Lymph nodes located subaortic and para-aortic lateral to the ligamentum arteriosum. Superior boundary is the lower margin of the aortic arch. Ventral boundary is the pulmonary artery, distal boundary the left main bronchus.
**9** Subcarinal lymph nodes	• Lymph nodes located caudal to the carina of the trachea. The lateral boundaries are the extended line of both lateral margins of the trachea.
**10(L/R)** Upper mediastinal paraesophageal lymph nodes	• Dissection of the lymph nodes located around the upper thoracic esophagus. From the thoracic aperture until the trachea bifurcation.
**Lower thoracic lymph nodes**	
**11(L/R)** Middle mediastinal paraesophageal lymph nodes	• Lymph nodes located around the middle thoracic esophagus. From the tracheal bifurcation to the caudal margin of the inferior pulmonary vein.
**12(L/R)** Lower mediastinal paraesophageal lymph nodes	• Lymph nodes located around the lower thoracic esophagus. From the caudal margin of the inferior pulmonary vein to the esophagogastric junction.

TIGER (1st edition)	
**Number and Name**	**Borders lymph node station**
**13L** Left pulmonary ligament lymph nodes	• Dissection of the lymph nodes within the left inferior pulmonary ligament.
**13R** Right pulmonary ligament lymph nodes	• Dissection of the lymph nodes within the right inferior pulmonary ligament.
**Abdominal lymph nodes**	
**14L** Left paracardia lymph nodes	• Lymph nodes located immediately adjacent to the gastroesophageal junction, including those along the esophagocardiac branch of the left subphrenic artery.
**14R** Right paracardia lymph nodes	• Lymph nodes located immediately adjacent to the gastroesophageal junction, including those along the first branch of the ascending limb of the left gastric artery.
**15** Left gastric lymph nodes	• Lymph nodes along the course of the left gastric artery. Superior boundary is the caudal border of the first branch of the ascending limb of the left gastric artery. Proximal boundary is the origin of the left gastric artery.
**16** Celiac trunk lymph nodes	• Lymph nodes located around the celiac trunk. Dorsal boundary is the aorta; ventral boundary is the origin of the left gastric artery.
**17** Splenic artery lymph nodes	• Lymph nodes from the origin of the splenic artery along its course alongside the pancreatic tail, including those adjacent to the splenic artery distal to the pancreatic tail, and those on the roots of the short gastric arteries and those along the left gastroepiploic artery proximal to its 1st gastric branch.
**18** Common hepatic artery lymph nodes	• Lymph nodes from the origin of the common hepatic artery along its course until the division into the gastroduodenal and proper hepatic artery.
**19** Hepatoduodenal ligament lymph nodes	• Lymph nodes along the proper hepatic artery and along the portal vein in the caudal half between the confluence of the right and left hepatic ducts and the upper border of the pancreas.
